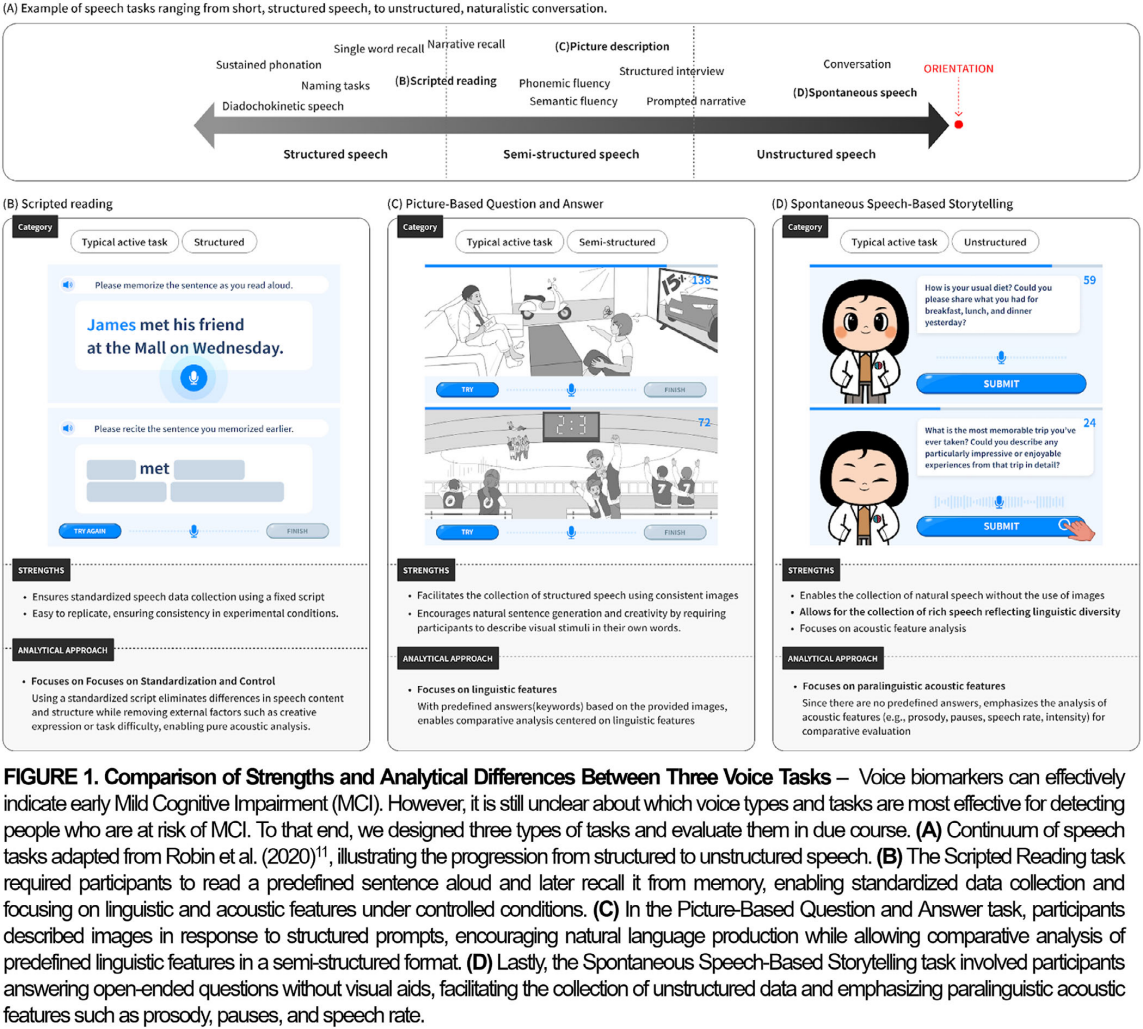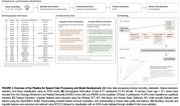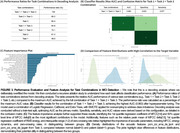# A Preliminary Investigation into How Free Speech Tasks Help Detect People Who Are at Risk of Mild Cognitive Impairment

**DOI:** 10.1002/alz70859_102772

**Published:** 2025-12-25

**Authors:** So Yoon Park, Ju Hyun Lee, Whani Kim, Hyun Jeong Ko, Byung Hun Yun, Jin Sung Kim, Jee Hang Lee, Geon Ha Kim, Jinwoo Kim

**Affiliations:** ^1^ HAII Inc., Seoul Korea, Republic of (South); ^2^ HCI Lab, Yonsei University, Seoul Korea, Republic of (South); ^3^ Graduate School of AI and Informatics, Sangmyung University, Seoul Korea, Republic of (South); ^4^ Department of Human‐Centred AI, Sangmyung University, Seoul Korea, Republic of (South); ^5^ Institute for Advanced Intelligence Study, Daejeon Korea, Republic of (South); ^6^ Ewha Medical Research Institute, Ewha Womans University, Seoul Korea, Republic of (South); ^7^ Neurology, Ewha Womans University Mokdong Hospital, Ewha Womans University College of Medicine, Seoul Korea, Republic of (South)

## Abstract

**Background:**

Voice biomarkers can effectively indicate early Mild Cognitive Impairment (MCI)1‐10. This study evaluates how each speech type performs in screening through active tasks. We also examine which combinations of these tasks best detect MCI.

**Method:**

We designed three tasks to that end: Scripted Reading (Task 1) and Picture‐Based Question and Answer (Task 2) for structured speech, and Spontaneous Speech‐Based Storytelling (Task 3) for semi‐structured speech (Figure 1). We collected 129 speech samples from 21 participants. Using Recursive Feature Elimination with Cross‐Validation (RFECV), we selected 32 key features from over 1,700 acoustic and linguistic ones for classification (Figure 2). We framed the evaluation process as a combinatorial problem where y=f(Task1 Task 2 Task 3). Here, y indicates whether a person is at risk of MCI, and f represents the predictive model that we trained with the speech samples.

**Result:**

The decoding analysis revealed that the combination of Task_1, 2 and 3 achieved the highest AUC performance (AUC 0.963; 100% ratio). Relative to this maximum performance, the combination of Task_2 + Task_3 achieved 0.869 (90.2%), followed by Task_3 0.822 (85.4%), and Task_1 + Task_3 0.817 (84.8%; Figure 3A). Among these, using all tasks resulted in the best classification

**Results:**

an AUC of 0.963, specificity of 0.633, and sensitivity of 0.977 (Figure 3B). The feature importance analysis revealed that 1st quantile regression coefficient of MFCC[14] and 50% upper level time of MFCC delta[9] were the most significant features contributing to the classification model (Figure 3C). Lastly, the acoustic features derived from Task_3, pcm_zcr_sma_de_lpc4 (representing the fourth coefficient of linear predictive coding; LPC) and pcm_zcr_sma_de_lpgain (reflecting the signal‐to‐noise ratio based on energy distribution), showed clear differences between normal and patient groups (Figure 3D).

**Conclusion:**

Among various task combinations, the combination of Task_1+Task_2+Task_3 consistently achieved the best results, with Task 3 being included in all high‐performance combinations. Feature importance analysis and target variable distribution further emphasized the greater contribution of acoustic features compared to linguistic features in classification performance.